# New Jersey State Veterinary Society

**Published:** 1891-03

**Authors:** A. T. Sellers

**Affiliations:** Secretary


*New Jersey State Veterinary Society.*—The second annual meeting of the New Jersey State Veterinary Society was held in Saenger Hall, city of Newark, on Thursday February 5, 1891. Meeting called to order promptly at 2 P.M. The President, Dr. E. Loblein occupying the chair. On roll call the following members answered to their names: Drs. Autenreith, Corlies, Hopkins, Krowl, Loblein, Lowe, Pacock, Sattler and Sellers. Minutes of last meeting were read and approved. It was regularly moved and seconded that the application of Dr. Dorney be laid on the table, carried. Moved and seconded that the secretary communicate with the Dean of the New York Veterinary College and find whether Dr. Otto Von Lang is a graduate of said college, carried. Dr. Hopkins proposed the following Veterinarians for membership, Drs. T. H. Ripley, E. R. Ogden, E. C. Batten and W. F. Harrison. The applications were referred to the Board of Censors for action. Dr. Lowe, on behalf of the United States Veterinary Medical Association extended to the New Jersey State Veterinary Society an invitation to attend their next meeting to be held in Washington, D. C., September next. On motion the invitation was accepted. On motion the bill presented by the secretary for printing, postage, etc., was ordered paid.

The use of sulphate of strychnia by hypodermic injection in Purpura Haemorrhagia was the subject of lengthy remarks by Dr. Hopkins, of Newark, who claimed that in one-eighth grain doses he has had remarkable success; before using this treatment his percentage of loss was great, but since using strychnia he has cured every case that he treated with it. He advocates the hypodermic injection of strychnia in one-eighth grain doses, every four hours, injected into the healthy tissues continued as soon as the case will permit with tonics, given by the mouth, iron, cinchona, gentian, etc., he stated cases that apparently were in the last stages of the disease which were completely cured by his treatment; in cases where the disease was just making its appearance, the disease was checked almost immediately. In fact in every case that the treatment was used, the patient improved as soon as the treatment was commenced.

Several cases of luxation of the patella due to rhino adenitis were reported, causing considerable discussion, the luxation was considered to be due to muscular relaxation, the case being exceedingly rare. Dr. Krowl gave the symptoms of a very interesting case of Melanosis in a black mare, he stated the impossibility of making a correct diagnosis, owing to the fact that the only place of the pigmentary deposit was in the kidneys, the kidneys degenerated, and formed abscesses which broke in the abdominal cavity causing death.

On motion it was agreed to hold the annual meeting in New Brunswick, adjourned.

A. T. SELLERS, D. V. S., *Secretary.*

